# Active Player Modeling in the Iterated Prisoner's Dilemma

**DOI:** 10.1155/2016/7420984

**Published:** 2016-02-18

**Authors:** Hyunsoo Park, Kyung-Joong Kim

**Affiliations:** Department of Computer Science and Engineering, Sejong University, 209 Neungdong-ro, Gwangjin-gu, Seoul 05006, Republic of Korea

## Abstract

The iterated prisoner's dilemma (IPD) is well known within the domain of game theory. Although it is relatively simple, it can also elucidate important problems related to cooperation and trust. Generally, players can predict their opponents' actions when they are able to build a precise model of their behavior based on their game playing experience. However, it is difficult to make such predictions based on a limited number of games. The creation of a precise model requires the use of not only an appropriate learning algorithm and framework but also a good dataset. Active learning approaches have recently been introduced to machine learning communities. The approach can usually produce informative datasets with relatively little effort. Therefore, we have proposed an active modeling technique to predict the behavior of IPD players. The proposed method can model the opponent player's behavior while taking advantage of interactive game environments. This experiment used twelve representative types of players as opponents, and an observer used an active modeling algorithm to model these opponents. This observer actively collected data and modeled the opponent's behavior online. Most of our data showed that the observer was able to build, through direct actions, a more accurate model of an opponent's behavior than when the data were collected through random actions.

## 1. Introduction

Understanding one's opponents is very useful when playing games. In many games, each player tries to figure out his or her opponents' hidden beliefs, desires, and intentions to maximize his or her reward. However, this is difficult in many cases because this piece of information is often hidden by the opponents. Instead, each player can usually only infer other players' internal states based on observable information such as behavior. This discussion raises questions about how players can understand one another and there are many possible explanations for the development of such understanding [1, 2]. However, in the present paper, we consider only player modeling, techniques/methods to build models that can predict/infer player's future behaviors, as an approach to understanding opponents [3]. Usually, it uses the player's past behavior data. First, this approach is simple and effective and can accommodate many techniques and styles of implementation based on the data. These methods can be easily used for player modeling, and the development of an accurate player model may enable us to infer a player's current inner state, predict his/her future actions, and figure out the reason for current actions.

The iterated prisoner's dilemma (IPD) [4] is among the games in which player modeling is important. This mathematical game is well known in the domains of economics, international politics, and artificial intelligence (AI). When playing the IPD, the ability to predict the future action of one's opponent is the most important contributor to maximizing one's own benefit. Generally, the creation of a precise model to predict the future action of an opponent is sufficient to win this game, and several studies have examined opponent modeling in the IPD [5].

Application of the modeling technique requires the prior collection of sufficient data, which is difficult because it entails that a player should play the game to provide data with ground truth. Modeling techniques based on data are usually known as data mining [6], an approach that has been studied for the last few decades and successfully applied in various domains. The success of many of these applications requires considerable data. Thus, insufficient data render the performance of the model uncertain. Indeed, more sophisticated models with more features require more data. This phenomenon is known as the curse of dimensions [7]. The same problems arise in player modeling. Although the labeling of a future action is easy in the IPD (labeling a future action involves only recording it), the problem of a limited dataset remains.

The development of an active learning algorithm [8] is one approach to dealing with the scarcity of data. This approach enables the collection of more informative data through online interactions/queries, and it usually increases the accuracy of the model with fewer data points compared with conventional (passive) learning algorithms. This approach can be applied in a straightforward manner to collect data related to the IPD, because interaction is an important element of this game. These interactions can form the basis for the active learning algorithm used to model the behavior of one's opponent.

In this paper, we propose an active learning method to be used for player modeling in the IPD game. Our approach is based on a query-by-committee (QBC) [9] and estimation exploration algorithm (EEA) [10]. QBC algorithms are among the active learning algorithms using multiple hypotheses (ensemble of models). EEAs use similar approach but originate in evolutionary computation. We conducted simulations to evaluate the performance of our approach. These simulations involved two players, an observer (learner) and an opponent. The observer played the game to model his/her opponent's behavior based on an active learning algorithm. The opponent was a typical player who was playing to obtain rewards. This approach involved 12 types of strategy. In this way, we comparatively evaluated the advantage offered by our method with regard to data collection. According to our results, our approach performed better in most cases. Over the course of a few games, it was able to build more accurate models than a random approach.

## 2. Related Research

### 2.1. Iterated Prisoner's Dilemma

In the original prisoner's dilemma, two players choose to cooperate (*C*) or to defect (*D*) and receive a reward or penalty based on their choice.  Table 1 presents the prisoner's dilemma's payoff table. If two players cooperate, each gets an intermediate reward (*R*: reward). However, if one defects when the other cooperates, the defector gets the maximum reward (*T*: temptation) and the cooperator gets nothing (*S*: sucker). If all players defect to avoid being penalized, the two players get the minimum reward (*P*: penalty). The payoff has to satisfy the conditions in (1), which prevents a strategy involving alternating between *C* and *D* from yielding an incentive and also motivates each player to play noncooperatively:

(1) T>R>P>S,R>12T+s.

It is usually in the best interest of both players to trust and cooperate with the other, because this strategy yields the maximum reward. However, there is always the risk that one's opponent will choose to defect. In this setting the cooperative action's expected reward is 1.5 ((3 + 0)/2) and defect action's expectation reward is 3.0 ((5 + 1)/2). Therefore, if each player is rational, defection is seen as the action that maximizes the expected reward. Furthermore, each player assumes the opponents are rational; in other words each opponent will defect to maximize an expected reward, and so each player should defect. This is the rational strategy in the original prisoner's dilemma.

The iterated prisoner's dilemma (IPD) is the elaborated version of this game. In this setting, all players can consider previous events as they choose their next action. Thus, each player can seek revenge for her/his opponent's defection and also take advantage of her/his opponent's cooperation. Under these conditions, a player seeking a cooperative situation should usually not be the first to betray the other. Indeed, we face many similar situations in real life. For this reason, many studies have used this game to understand many real-life phenomena [11, 12].

### 2.2. Player Strategies for the IPD

Many playing strategies can be used in the IPD, for instance, always cooperate (AllC) or defect, tit for tat (TFT), majority (Major), and Pavlovian strategies. However, no strategy wins against all other types of strategy, and all strategies are stronger or weaker against specific strategies. Therefore, game results depend on the players who participate [4, 13, 14]. Axelrod, a researcher who performed seminal work on the IPD, organized two tournaments that identified and enabled analysis of different strategies and their characteristics [13]. TFT, which was among the better strategies used in the first tournaments, and its variations produced good results. These types of strategy involve initial cooperative play, revenge in response to betrayal, and forgiveness when cooperation follows defection. Thus, a player cooperates in response to cooperative action, defects to defend against defection, and promotes cooperative play when the player who previously defected became cooperative. Strategies such as TFT do not work optimally against all other types of strategy, but they can avoid the worst situation (sucker). When an opponent is cooperative, these strategies reap the benefits of cooperation. In other cases, they avoid exploitation by the opponent.

Although TFT-style strategies work well against other strategies, many studies have tried to identify even better strategies. Such attempts to identify strategies can be formulated as a kind of search problem in which finding a better strategy is equivalent to finding a better solution. Evolutionary computation approaches (genetic algorithms, evolutionary strategies, etc.) are suitable for this kind of search problem and can also adapt to changes in the opponent's strategy. Following one tournament, Axelrod used an evolutionary algorithm to identify a strategy that was equal to or better than TFT [15]. This initial effort was followed by many other attempts to work on this problem based on evolutionary computation [5, 14, 16–20].

It has been a long research question to derive a strategy to be generalized well on an unseen opponent. It involves studies on learning algorithms, strategy representation, and analysis of strategies. For example, Fogel studied the conditions that promote the evolution of cooperative behavior using evolutionary programming, one of the evolutionary computation algorithms [16]. Nowak and Sigmund identified a Pavlovian strategy that was more robust than TFT [14]. Additionally, Beaufils et al. reported on a new gradual strategy [17], and Van Bragt et al. examined the selection scheme used in [18]. Indeed, the selection mechanism is one of the major contributors to the performance of evolutionary algorithms. Ashlock et al. investigated representations of agent strategies [20] by surveying various types of representation and investigating their underlying logic. They also proposed an opponent modeling tool known as a fingerprint, to compare complex strategies. Ishibuchi et al. studied the effect of representations in a 2D-grid world [21].

Several recent studies have examined the approach, which relies on understanding the strategy of one's current opponent. This understanding allows the player to predict his/her opponent's next action and choose the optimal action based on this piece of information. Li et al. examined this approach [22] using a simple rule-based strategy-identification mechanism for round-robin IPD competition. The player using this approach outperformed an opponent using well-known strategies. Gaudesi et al. discussed more sophisticated strategies involving inference techniques [5]. They used evolutionary computation to model the opponent's strategy as a finite state machine. During the game, the player using their approach built a player model for her/his opponent and used it to predict the opponent's future actions. According to their preliminary results, their method was able to accurately model TFT and perform well against other strategies. Recently, Gaudesi et al. propose opponent modeling method in [23]. They use evolutionary algorithm to model opponent behavior and use the best model to determine the next action. They use some heuristics and brute force simulation in order to find a next action with highest payoff.

### 2.3. Active Learning Algorithm

Active learning involves sampling data during the learning process instead of passively accepting all the available data. In other words, active learning assumes that an algorithm can collect more informative data by allowing selectivity. More informative data are selected with the use of an algorithm based on previously collected data and currently generated models. Through repeated use of these processes, we can collect informative data from a small number of cases.

From the perspective of an active learner (observer), a few pieces of data are labeled data, and there are considerable unlabeled data in the version space (possible configuration of data). When few labeled data are available, the learning algorithm can model the data, albeit imprecisely. Therefore, the observer can use the imprecise learned model to predict or label opponent behavior. Based on the predictions, it is possible to choose informative instances from the unlabeled data. In the next step, it queries the user or teacher in order to label the informative instances. Action learning repeats these two steps (modeling based on collected data and querying about informative data) and yields a more precise model with a smaller dataset.

Basically, active learning determines the most informative data in the current iteration based on uncertainty/disagreements. When the labeled data are available, the algorithm develops a model as a hypothesis. The basic active learning algorithm builds one model based on posterior probability [24], and uncertainty derives from its boundary (in a binary classification, the posterior probability is about 0.5). On the other hand, active-learning-using committees (ensembles) [8] apply many hypotheses, and disagreements reflect differences among models. This approach is known as the query-by-committee (QBC) algorithm.

The EEA [10] is similar to active learning. However, it originates from evolutionary computation, and it was developed to model complex nonlinear systems with little interaction (experiments). Like active modeling (modeling and query), it repeats the two steps of estimation and exploration. During the estimation step, it builds various types or structures of models using evolutionary computation. In the exploration step, it develops an informative query to improve current models based on differences among models. From an evolutionary perspective, this algorithm is among the coevolutionary algorithms that develop query and validate solutions simultaneously.

## 3. Active Modeling Framework for IPD

**Figure 1:**
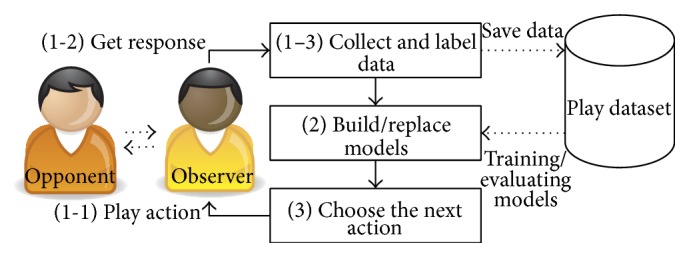
Overview of system.

In this section, we introduce our proposed active learning algorithm for modeling IPD players. This method hybridizes QBC and EEA. Particularly, our algorithm used an ensemble of diverse models for strategy representation and enhanced active learning algorithms for game player modeling.  Figure 1 presents an overview of our proposed method. The observer is the player that uses our proposed method. Getting a more immediate benefit is not the purpose of the observer. Instead it chooses an action for exploration of the opponent's decision process only. At each turn (opponent and observer engage in one action) of the game, the observer puts new data about the current game state to play into a dataset and determine the next action using active learning with the collected dataset.

The active learning algorithm consists of three parts: (1) playing the game and collecting data (play an action, get an opponent response, and label the data), (2) building models with the play dataset in the current turn, and (3) choosing the next action to obtain the game state with the greatest disagreement (uncertainty) in prediction. Our system models the opponent player's decision process during the game through these repeated three steps. We describe each step in detail and discuss how to apply it in an IPD game below.

### 3.1. Data Collecting and Label Data

**Figure 2:**
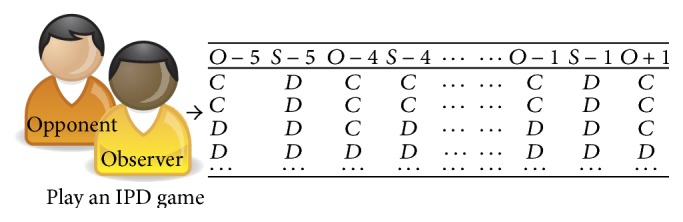
An example of data representation (*N* = 5).

In this part, this system models the behavior of the opponent based on collected play dataset. These data can be available before games or obtained by playing games. However, our system in this paper uses collected data during the current IPD game.  Figure 2 presents an example of the data representation. In each turn, the observer has information about the opponent's action in the current game state. We assume that all players select future actions based on their opponents' and their own previous *N* actions. For example, the attributes of each data instance are *O* − 5, *S* − 5, *O* − 4, *S* − 4,…, *O* − 1, and *S* − 1. *O* − *n* indicates an opponent's previous *n*th action and *S* − *n* refers to an observer's (self) *n*th action. Labeling of data instances (prediction of an opponent's next action) is represented as *O* + 1. The label can be easily assigned after an opponent engages in an action. Because this game has only two actions, cooperation and defection, basically all the values of the attributes are either *C* (cooperate) or *D* (defect). However, the values are invalid in the first *N* turns. In this case, we ignore the data because it represents a very small portion of the play dataset.

### 3.2. Modeling Labeled Data

The most important function of this part of the process is building various types of models with limited data. However, some learning algorithms are unable to build various models in such cases. As this is not good for our system, we used a bootstrapping approach (bootstrap aggregation) also known as bagging. This is the one of ensemble techniques in machine learning. It uses randomly generated data from the original dataset to avoid overfitting. When we built each model, we used (uniformly) randomly sampled data obtained from the entire dataset. Sampling is with replacement (one instance may appear multiple times). This approach allowed us to build a variety of models and was also employed to measure accuracy of each model. Since we can use current play dataset only, instead of an independent validation dataset, we use randomly sampled data for model validation too.

**Figure 3:**
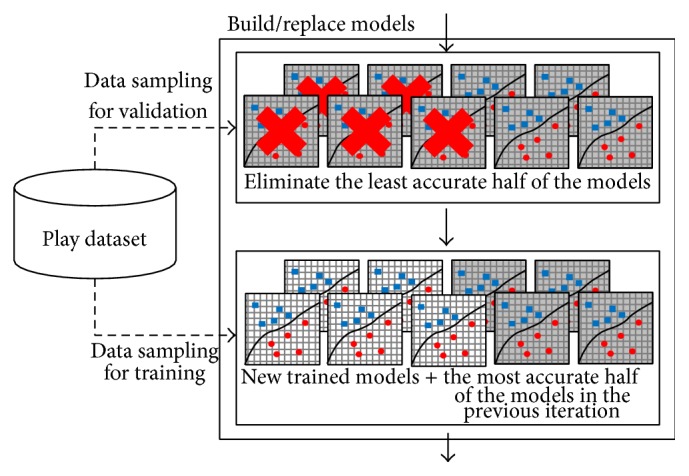
Building various types of models.

Figure 3 depicts the model ensemble building/training process. Our system contained *M* models (in experiments *M* = 50) trained by various types of machine learning algorithms. We used a decision tree (Cart), perceptron, support vector machine (SVM), *k*-nearest neighbor (*k*-NN), and naïve Bayes algorithm (NB) to build models. We use implementation of machine learning library scikit-learn [25]. Basically, we use default parameters, but we choose random parameters (e.g., kernel type for SVM,* k* for *k*-NN) for models, when the new models are built or the old models are replaced. There are two possible values in kernel types, linear or RBF (Radial Basis Function), and four values for* k* (1, 3, 5, and 7). These models can be replaced with new ones depending on their validation accuracy. In each game turn, only the most accurate half of the models remain in the model ensemble, and the least accurate half are replaced by new ones. For example, if there are Cart and perceptron models in the previous game and Cart models show relatively low performance in the current iteration, then the Cart models could be replaced with other types of models. When new models are built, one of five training algorithms and parameters is randomly chosen. The selection of algorithm and parameters was performed every time when a single new model is created. If the model needs data with real values (e.g., perceptron), then *C* is changed to 1.0 and *D* is changed to −1.0.

### 3.3. Choose the Next Action

The active learning algorithm provides several approaches to distinguishing the most informative data that should be labeled by user or teacher. For example, they are based on prediction uncertainty, disagreement of committee, change to the current model, and expected error reduction. Generally, finding an uncertainty area in the version space indicates the presence of informative data that should be used to improve the current models. The QBC algorithm is among those that identify uncertainty based on an ensemble of models. If there are various models with sufficient accuracy, the uncertainty space in the current models is the same as the disagreement among the models. In other words, we have to find the future game state with diverse predictions (showing disagreement in predictions) by models.

**Figure 4:**
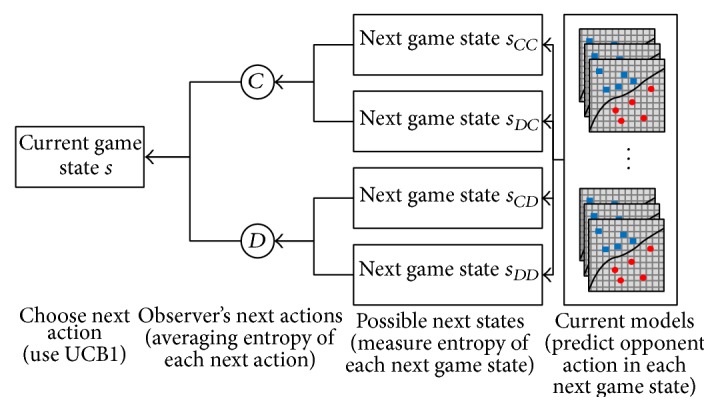
Choose next action based on disagreements.

Figure 4 shows the whole process of how our algorithm chooses the next action. Always there are two possible actions (*C* and *D*) and four possible following game states that depend on each player's next action. Our algorithm's purpose is to find the most informative data (game state and opponent's action) among these four states. In order to do this, it measures disagreements of current models in these four game states and maps these to each possible action. In other words, it measures each action's benefit in exploring the opponent's strategy. There are three things to consider, how to measure disagreements, how to map disagreements to each action, and how to combine disagreements and possibility of success in the data collecting policy. We describe this below:

(2) $$\begin{array}{c} H\left(\;X\;\right)=-\sum_{x\in \left\{C,D\right\}}p\left(\;x\;\right)\log p\left(\;x\;\right). \end{array}$$

Our system uses entropy [26] to measure disagreement. Entropy, which refers to information content in information theory, is one of the possible metrics for measuring disagreements. Equation (2) shows how it measures the entropy (*H*(*X*)). There are two possible opponent actions: *C* and *D* in the current game state. *p*(*x*) refers to the probability (ratio of prediction *x*) of prediction. For instance, three models predict *C* and the other eight models predict *D*; then *p*(*C*) = 3/11 and *p*(*D*) = 8/11. If all models in the current models have identical predictions of an opponent's future action, entropy is low (the minimum value is 0.0); however, when half the predictions favor *C* and half favor *D*, entropy is at its maximum. There are only two nominal symbols (*C* and *D*), and maximum entropy is 1.0. If we were dealing with conventional active learning application, we would have to search all conditions (game states) with the highest entropy values. However, it is enough to find possible future game states only in the game environments.

Probably, it could be easy to get data that the algorithm queried if it were not a game. Common active learning algorithms try to find which data is the best to increase the current model's performance and to label it by query to oracle (e.g., human). In this case, the oracle is cooperative with the algorithm and therefore the data collecting process is relatively easy. However, in game sceneries like IPD, it is different. In IPD, there are no reasons for the opponent player to cooperate with data collecting to reveal their strategy. Therefore, the active learning algorithm in the game considers the possibilities of the target data collecting success as well as finding informative data.

The reason for the difficulties in data collecting is that the observer cannot control the opponent's action. For instance, if the current game state is *CC* | *CC* | *CC* (considering only the past three turns, e.g.) and the state with maximum disagreement is *CC* | *CC* | **D** **D**, then the observer and the opponent should do *D* to collect informative data. However, if the opponent chooses *C* only, the observer gets *CC* | *CC* | **C** **D** only instead of *CC* | *CC* | **D** **D**:

(3) $$\begin{array}{c} UCB1={\overset{-}{x}}_{j}+\sqrt{\frac{2\ln n}{n_{j}}}. \end{array}$$

To tackle this problem, we propose using UCB1 (Upper-Confidence Bound). Originally UCB1 is used to handle exploration-exploitation dilemmas. UCB1 equation (3) includes two terms; each term encourages exploitation and exploration. Originally, the exploitation term (left) reflected (action* j*'s) expectation of reward, and the exploration term (right) reflected the number of trials (*n*: total number of actions, *n*_*j*_: number of action* j*). It increases when the number of actions *j* is too low relative to other actions. It reflects ignorance of action *j* in the current game state. As a result, UCB1 values increase when action* j*'s expectation value is high or/and number of trials is low.

In this paper, we use UCB1 in an uncommon way (balancing exploration and exploitation). The observer uses UCB1 to balance two types of exploration: exploration by disagreements of models and exploration by number of trials. Both reflect uncertainty or ignorance of our knowledge, but they have different mechanisms to measure it. The exploitation term (the left term of UCB1) is the entropy of the disagreement instead of the reward or benefit. If the observer gets the target data successfully and disagreement is resolved, then we think the observer gets the same amount of information as the target data's entropy. And the right term in the UCB1 determines the number of trials; therefore the observer tries other actions; even though it tries many times it cannot collect target data:

(4) UCB1C=HCC+HDC2+2ln⁡nnc,UCB1D=HCD+HDD2+2ln⁡nnD.

In order to apply this concept, we use (4). It is almost the same as the original UCB1; we only change the left term for our application. The left term is the average entropy of the possible future states' entropies. For example, if the current game state is *CC* | *CC* | *CC*, then there are four possible following game states (*CC* | *CC* | **C** **C**, *CC* | *CC* | **C** **D**, *CC* | *CC* | **D** **C**, and *CC* | *CC* | **D** **D**) based on the observer and opponent's next action. Since the observer cannot control the opponent's action, the following game states will be (*CC* | *CC* | **C** **C** and *CC* | *CC* | **D** **C**) or (*CC* | *CC* | **C** **D** and *CC* | *CC* | **D** **D**) based on the observer's action. If an observer selects *C* then the game state will be *CC* | *CC* | **C** **C** or *CC* | *CC* | **D** **C**, but it depends on the opponent's action as to which one becomes the real game state. Therefore, we use the average of both *H*_*CC*_ (entropy in *CC* | *CC* | **C** **C**) and *H*_*DC*_ (entropy in *CC* | *CC* | **D** **C**) to get the observer action's results. When we get the UCB1 value, the observer chooses the next action. If UCB1_*C*_ is greater than UCB1_*D*_, the observer chooses *C*. Otherwise it chooses *D*.

## 4. Experiments

In the current section, we describe our experiments and their results. In these experiments, the observer played games with the opponent with the goal of building a prediction model that can predict the AI player's next action.

### 4.1. Player Types (Strategies)

We used 12 types of players for our experiments; these are summarized in Table 2. There were six unique strategies and six variations: AllC, ADP, TFT, FTR3, Major, and Pavlovian are famous strategies used in the IPD. The NoisyTFT, TF2T, and Adaptive TFT are well-known variation of TFT, and Major 5 is a special variation of Major.

(1) AllC refers to a strategy of always cooperating. (2) CCD repeats *C*, *C*, and *D* actions. AllC and CCD do not change their decisions depending on opponent behaviors. These are very simple strategies.

(3) TFT is the most popular strategy in IPD, involving initial cooperation and subsequent mimicking of the opponent's last action. Thus, one cooperates when one's opponent cooperates, one seeks revenge when one's opponent defects, and one forgives (cooperates) when one's opponent moves from defection to cooperation. This is usually a good strategy for all types of players. (4) NoisyTFT is a variation of TFT, but is more complex (i.e., more difficultly build a model) [27]. Actions change with a probability of 0.1. (5) TF2T is similar to TFT, but it responds with defect only when the opponent chooses defect two times in a row. (6) ATFT is an adaptive version of TFT. It has variable *w* that changes dependence on opponent actions. If opponent more cooperates *w* will increase; otherwise it will decrease. ATFT decides the next action based on *w*.

(7) ADP is a basic adaptive strategy. It starts with five consecutive *C* and five consecutive *D*. After that, it chooses an action with high payoff.

(8) Players following the Major strategy make decisions based on all the previous actions of the opponent. If the opponent cooperated more than she or he defected, then a player following this strategy would cooperate. Otherwise, she or he would defect. Because our system considered a maximum of five previous actions, it was difficult to build a model based on this strategy. (9) Thus, we modified the Major strategy to consider only five past actions, naming it “Major 5.” (10) FS cooperation depends on *p* (probability of opponent's cooperation). It is similar to Major except it cooperates with probability *p* (initial *p* is 0.5).

(11) The Pavlovian strategy involves repeating the last action if it yielded a high return; otherwise, it involves engaging in a new action. In other words, a player following this strategy cooperates when the opponent's behavior in the last round was the same as her or his own. Otherwise, the player chooses defects.

(12) FRT3 (fortress 3) tries to recognize play pattern using *D*, *D*, and *C* action sequences. If an opponent plays the same pattern, it selects *C* or otherwise *D*.

### 4.2. Experimental Results and Analysis

**Figure 5:**
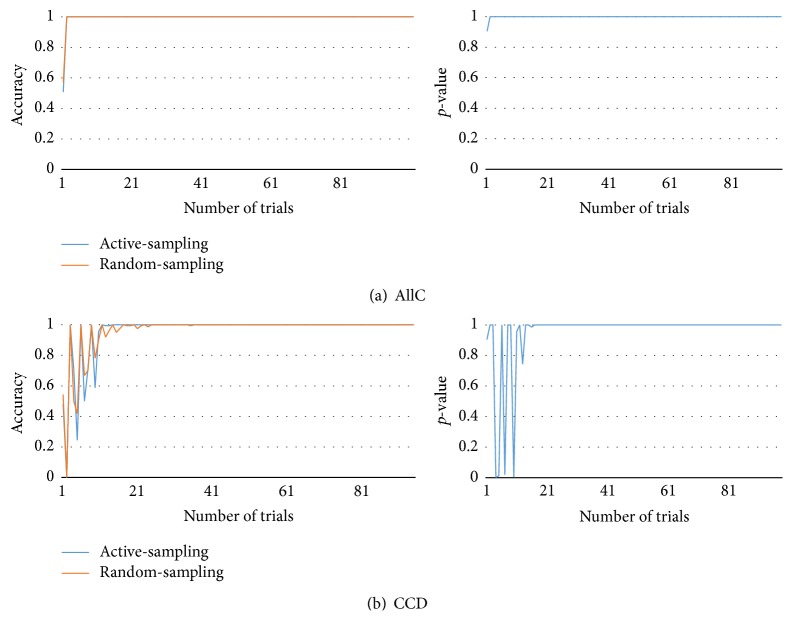
Test accuracy and *p*-value of active-sampling and random-sampling (AllC and CCD).

Figure 5 compares the results of the proposed method with those of the random-sampling approach. Because our proposed method involves the active collection of data, we named it “active-sampling.” In contrast, the other method does not use active-sampling based on disagreements; instead, it chooses the action randomly. In other words, it collects data randomly. Therefore, we named it the “random-sampling” approach. Because it also uses an ensemble of models, it also offers advantages in terms of prediction of the ensemble techniques. In each game, each player was presented with 100 opportunities to choose an action (100-turn game), and the observer (active-sampling and random-sampling) built models using data contemporaneously collected. Active-sampling observer maintains 50 models of ensembles. After a game ends, collected data were discarded. They were not used for the next game. In this study, the algorithm looked back at the last five time steps. Because the number of steps controls the number of inputs of the opponent model, the parameter has relation with the model complexity. If the step number is too small, the information is not enough to be modeled. On the other hand, it is too complex to model if the history looks back too far. We set the parameter as five because it is the smallest one derived from initial testing. It is also possible to control the number of steps to look ahead but it also increases the uncertainty of estimation.

**Figure 6:**
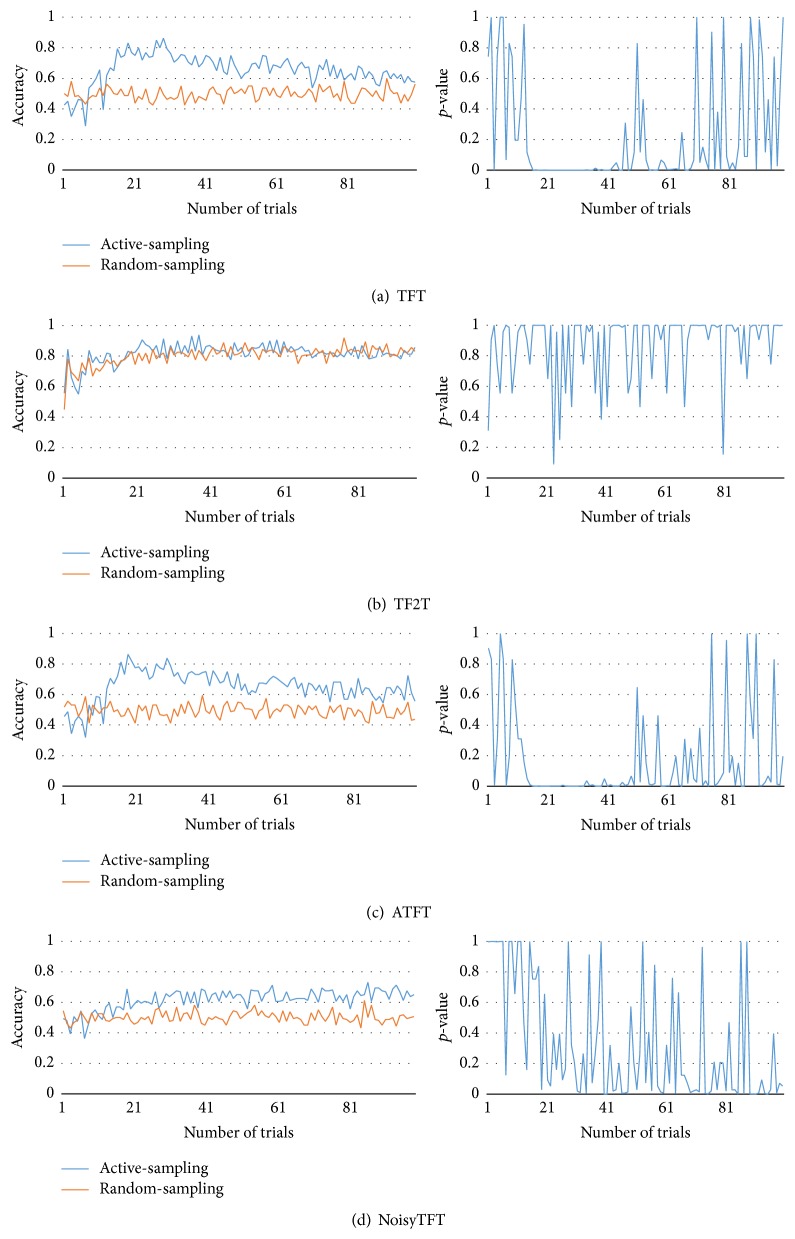
Test accuracy and *p*-value of active-sampling and random-sampling (TFT, TF2T, ATFT, and NoisyTFT).

**Figure 7:**
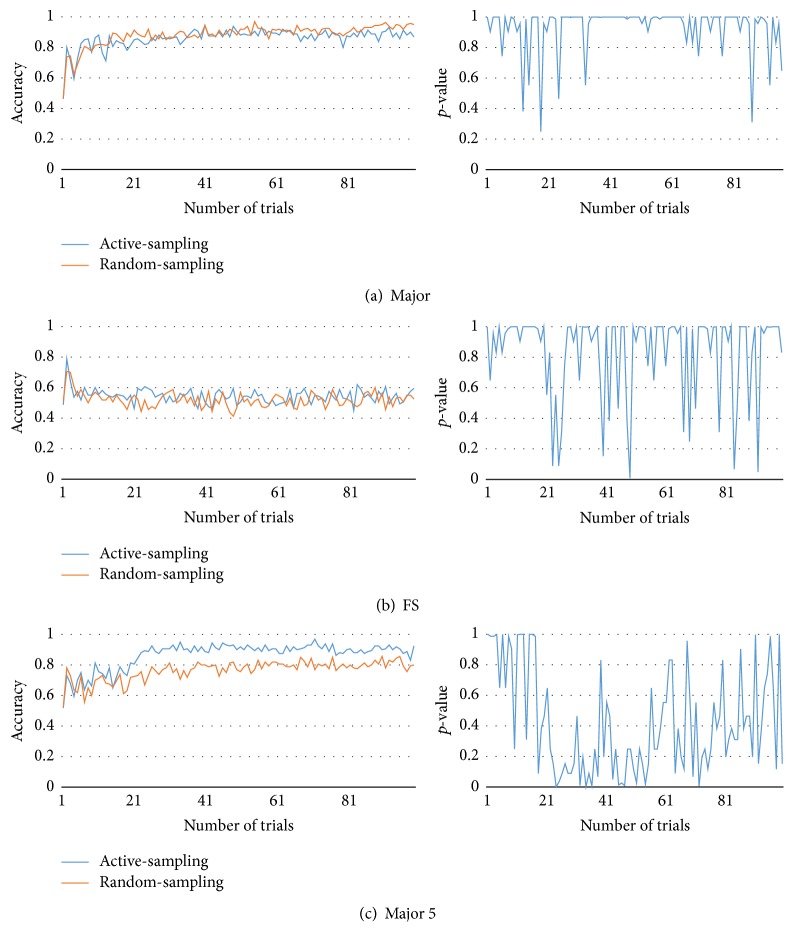
Test accuracy and *p*-value of active-sampling and random-sampling (Major, FS, and Major 5).

**Figure 8:**
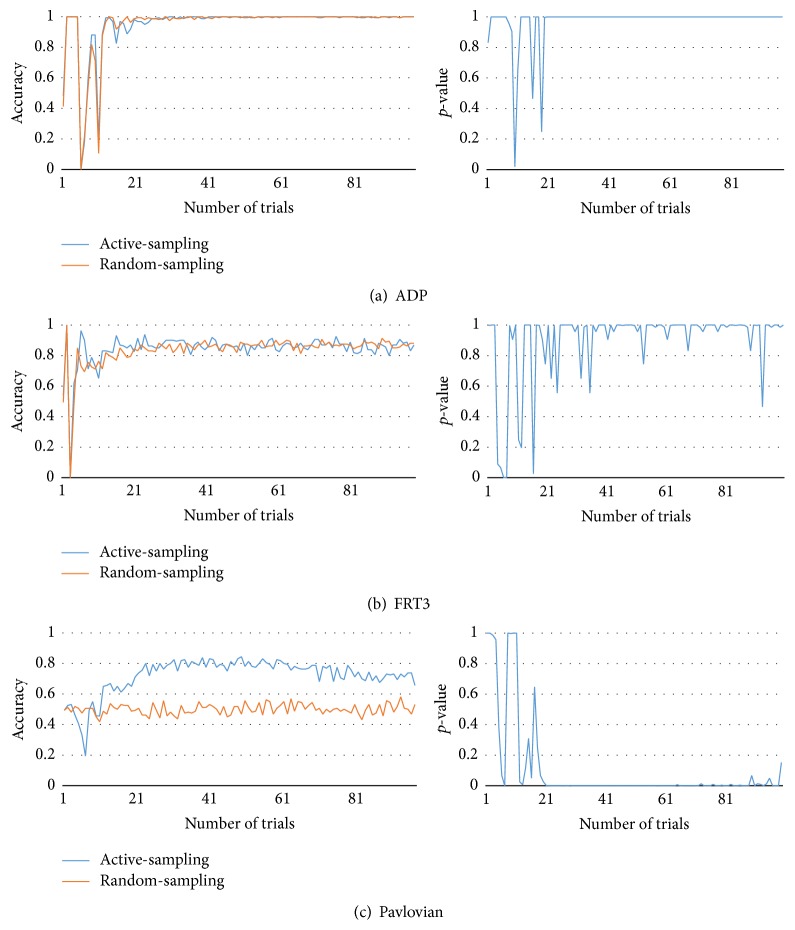
Test accuracy and *p*-value of active-sampling and random-sampling (ADP, Pavlovian, and FRT3).

In order to test the performance of the models, we generated test data. Since there are only 1024 (2^10^) game states, we can get all possible game play data easily and record all responses of opponent types in all possible game states. At each turn, we tested the prediction model (active-sampling and random-sampling) using the test data. The prediction model's outcome is the majority voting of models. However, we could not ascertain the actual opponent action of those following the NoisyTFT and the Major strategies. In NoisyTFT, we use TFT testing data. In Major, we generated testing data in online manner. Figures 5, 6, 7, and 8 present the average accuracy (over 100 experiments) under each condition.

We show accuracy changes in left panel and *p*-value changes in right panel. Accuracy shows the ratio of correct prediction of each approach at certain time. And *p*-value (we used two-sample Kolmogorov-Smirnov test) shows statistical significance of the accuracy difference.  Figure 5(a) presents the results under the AllC condition. Because they were very easy to model, the active- and random-sampling approaches were completely modeled after one action. (b) CCD shows similar results but it took some trials to model completely. After 20 trials, *p*-value of active and random sample is almost 1.0; it means that there is no difference between the two approaches.

Figure 6 shows the results of the TFT and its variants (TF2T, ATFT, and NoisyTFT). In (a) and (c), active-sampling approach yielded more accurate predictions between trails 20 and 70. On the other hand, random-sampling approach was barely predicting the opponent's action. Also *p*-values are quite small (under 0.1) in this period. However, benefit of active-sampling gradually disappeared after 70 trails. (b) shows the worst results. In (b), active- and random-sampling approaches show almost the same accuracy. However, against noisy opponent (d), active-sampling approach shows better results again. It yielded better prediction and *p*-values are relatively small.

Figure 7 shows the results of Major, FS, and Major 5. In (a), unlike the data in other cases, it reports the relative success of random-sampling. For Major strategy, random-sampling produced slightly better predictions than the active-sampling approach. In (c), active-sampling approach yielded better prediction accuracy with lower *p*-value between 20th and 60th trials.

Figure 8 shows experimental results of ADP, Pavlovian, and FRT3. In (a) and (b), active- and random-sampling approaches show almost same results. Both approaches can model these strategies sufficiently. In (c), active-sampling approach yielded almost 30% higher accuracy and its *p*-value remains in almost zero after 20th trial.

According to the experimental results, our proposed active-sampling method outperformed the random-sampling method in TFT, NoisyTFT, Major 5, and Pavlovian. In other cases, both approaches show almost same results. The accuracy difference depends on the complexity of the opponent's strategy. For example, in the case of the AllC, the simplest strategy, no differences were observed between the active- and random-sampling approaches. Because this strategy is too simple to form the basis of models, there are no possible factors that can lead to differences between the approaches. In contrast, major differences in the accuracy of more complex strategies were observed.

Table 3 presents differences in the average accuracy between active- and random-sampling in all games (100 game turns × 100 experiments = 10,000). We pick six representative strategies from 12 strategies. At the start of the game, these differences were very small, but they increased after 20–30 turns. The difference in training accuracy was relatively small (under 0.07) during play, indicating the absence of major differences in the modeling ability of the two approaches. The training accuracy measures used obtained data in the current game (validation process using randomly sampled training data). However, when testing data (all possible game play datasets) were used, the difference increased. With the exception of the AllC and Major conditions, the differences in the accuracy of the approaches exceeded 0.10. The largest difference was observed with regard to the Pavlovian approach (0.205), which may indicate that the random-sampling approach suffers from an overfitting problem. On the other hand, our proposed active-sampling method can prevent such overfitting.

There was only one case in which the results of our approach were worse than those of random-sampling. When modeling the Major strategy, an active-sampling approach yielded worse results than did a random-sampling approach. The reason for this phenomenon involves our representation of data, because our representation was a simple 10-length vector of each player's past five actions. Therefore, an observer can consider only the past five actions, which is not sufficient for building a model of the Major strategy. On the other hand, the experiments using Major 5 (a version of Major modified for our experiments) reflected better performance. Because Major 5 considers only the opponent's five prior actions, it fits our data representation.

**Figure 9:**
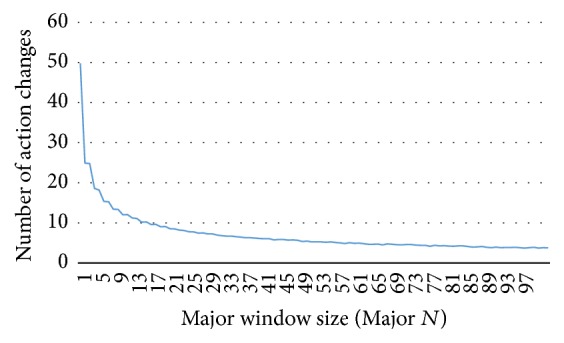
Relationship between window size and number of action changes (avg. of 1,000 games).

However, the random-sampling approach used in the Major and Major 5 cases produced different results. Although the random-sampling player chooses randomly, these choices could have become biased toward one action (*C* or *D*) during a lengthy game.  Figure 9 shows the relationship of window size and number of action changes in a game fitting a Major strategy against a random one. When the window size was 100 (the original Major strategy that considers all opponent actions), the opponent following the Major strategy changed actions (*C* → *D* or *D* → *C*) on fewer than four occasions. This was easy to model because the opponent's behavior was simple and rarely changed. However, when the window size decreased, the number of action changes increased exponentially, as was the case with Major 5. Therefore, it was difficult to model the Major in the absence of an appropriate learning algorithm and a sufficient dataset. Additionally, we can understand TFT as Major 1 model, which is more difficult to model using random-sampling.

## 5. Conclusion and Future Directions

Recently, active learning has emerged as one of the most promising machine learning techniques. This type of algorithm can choose data that can improve currently trained model(s), and it can use this mechanism to build a model that performs better compared with the conventional (passive) machine learning algorithm. Generally, these types of algorithms rely on interactive circumstances to establish an archive that is used to improve performance. Therefore, a game such as IPD is suitable for this approach. In this paper, we proposed active learning methods for the IPD, because this approach collects data efficiently in this game scenario.

In this study, we introduced the hybrid of QBC and EEA using ensembles of diverse models trained with bootstrapping techniques. It adaptively searches for the best ensembles to model opponent's strategy from the interaction with opponents. Because the IPD problem has special constraint on the manipulation of opponent, the uncertainty was considered in the equation to select the next action. The active modeling of opponent' strategy can increase the accuracy incrementally by collecting useful play history. To show the usefulness of this approach, the proposed method was tested with 12 strategies. In simple strategies, there is no significant difference between active- and random-sampling approaches, but there is meaningful performance gain for some complex opponent strategies. In case of unsuccessful results, in-depth study revealed the condition that the active learning is not working well.

In this paper, we proposed an active-sampling method for game environments. Although we used only the very simplest game, IPD, our approach can also be applied to all kinds of interactive scenarios. In game AI community, this work is early stage of discussion on the use of active learning for video games [28]. Therefore, future research should apply this approach to other games or interactive scenarios. Because an active learning approach can obtain data effectively (reduce overfitting), it reduces the human-user effort involved in data collection. Although our experiments involved an AI opponent player, it would be useful to test our hypotheses using a human user in the future.

Additionally, research regarding the effect of data representation and learning algorithms should employ other applications. As shown in the Major strategy experiments, data representation is very important. Although active-sampling can obtain data effectively, it cannot compensate for unfitted data representations in the same learning algorithm. We used a conventional machine learning algorithm in this study, but learning algorithms are dependent on data representation. Also, it is possible to improve learning efficiency using model complexity measure. Active learning algorithms like QBC, EEA, and our proposed method's efficiency rely on divergence of models. If we evaluate each model in ensemble using accuracy as well as model complexity, we can prevent each model in the ensemble from being too similar.

Gaudesi et al. used evolutionary computation to model opponent strategy with nondeterministic finite state machine (FSM) [23]. Similarly, it models opponents without any prior knowledge in an online manner based on the interactions from games. However, in our study, we purposely select the next action of player to actively model opponents. Because the manipulation of opponent is not always successful in the game of IPD, the efficiency of modeling is depending on the quality of data collection. Also, the choice of representation has impact on the outcomes of research. For example, [23] uses nondeterministic FSM but this study uses an ensemble of multiple models. It might be more useful to include the FSM into one of member models in the ensemble. It is also important to design common benchmarking framework for active learning problems in the IPD context.

The IPD agent ideally needs to solve the two problems at the same time: accurate modeling opponents and maximizing outcomes. However, the current state of the art in IPD research focuses on either opponent modeling or maximizing outcomes. In this study, we only focus on using active learning mechanism for modeling opponents in IPD. As a future work, it is highly desirable to incorporate the cost term (the outcomes acquired from the game playing) together with the modeling accuracy. To optimize both terms, to use multiobjective optimization algorithms to discover multiple Pareto optimum instead of this paper's single-objective genetic algorithms is recommended.

In machine learning, there have been several ways to avoid the overtraining and maximize generalization ability on unseen dataset. It is one of the widely used techniques to consider the complexity term together with accuracy. Additionally, it is also desirable to use multiple diverse models and combine them to generalize well (known as “ensemble” technique). In this study, we adopted the ensemble approach to increase generalization ability with “bootstrapping” techniques specialized to increase diversity of models. As a future works, it is useful to introduce the model complexity term (penalty terms for the number of parameters) to the model comparison.

## Figures and Tables

**Table 1:** Payoff table (player 1's payoff comes first).

	Decision	Player 2	
		Cooperate	Defect
Player 1	Cooperate	*R* = 3, *R* = 3	*S* = 0, *T* = 5
	Defect	*T* = 5, *S* = 0	*P* = 1, *P* = 1

*R*: reward, *S*: sucker, *T*: temptation, *P*: penalty.

**Table 2:** Player types.

Player type	Description
AllC	(i) Always cooperate

CCD	(i) Repeat *C*, *C* and *D*

TFT	(i) Mimic opponent's previous action (ii) The first action is cooperation

NoisyTFT	(i) Almost the same as TFT (ii) Action changes with a 10% probability

TF2T	(i) *C* after two opponents consecutive cooperation (ii) *D* after two opponents consecutive defections

ATFT	(i) Adaptive TFT (*w* = 0.5, *r* = 0.99) (ii) If *w* ≥ 0.5, then *C* or otherwise *D*

ADP	(i) Test *C*, *C*, *C*, *C*, *C* and *D*, *D*, *D*, *D*, *D* when game starts (ii) Choose an action based on payoff

Major	(i) Follow opponent's major actions during the entire game (ii) The first action is cooperation

Major 5	(i) Similar to Major (ii) Considers only the five previous actions

FS	(i) Defect with probability *p* (initial value 0.5) (ii) *p* is probability for opponent defection

Pavlovian	(i) Cooperate if my action was the same as that of my opponent in the last turn (ii) Otherwise, defect

FTR3	(i) Use a *D*, *D* and *C* pattern to recognize opponent's strategy (ii) If opponent's response is the same as the pattern, cooperate

**Table 3:** Differences in average accuracy of active- and random-sampling approaches.

Player type	Difference in training accuracy	Difference in testing accuracy
AllC	0.001	0.001
TFT	0.061	0.150
NoisyTFT	0.018	0.123
Major	−0.014	−0.081
Major 5	0.019	0.101
Pavlovian	0.042	0.205

Although the differences in the accuracy of the active- and the random-sampling approaches were minimal in the training, large differences were observed in the testing. This implies that active-sampling may prevent overfitting (difference = accuracy of active-sampling − accuracy of random-sampling).
